# Two successful natural pregnancies in a patient with severe uterine prolapse: A case report

**DOI:** 10.1186/1752-1947-5-459

**Published:** 2011-09-14

**Authors:** Davide De Vita, Salvatore Giordano

**Affiliations:** 1 Department of Obstetrics and Gynaecology, Santa Maria della Speranza Hospital, via Fiorignano, Battipaglia, 84091, SA, Italy; 2 Department of Surgery, Division of Plastic Surgery, Turku University Hospital, OS 299, PL 52, 20521, Turku, Finland

## Abstract

**Introduction:**

Uterine prolapse is a common gynecologic condition that is rare during or before pregnancy. We report an exceptional case of two pregnancies in a totally prolapsed uterus.

**Case presentation:**

A 36-year-old Caucasian woman with a history of uterine prolapse presented with pregnancy. A vaginal pessary was applied to keep her uterus inside the pelvis after manual reposition. The pessary was removed at the 24th week. The gravid uterus persisted in the abdominal cavity because of its increased volume.

**Conclusion:**

Our case shows that pregnancy during uterine prolapse is possible and that careful assessment is required to prevent complications during delivery. According to our experience, an elective caesarean section near term could be the safest mode of delivery.

## Introduction

Uterine prolapse is a common gynecologic condition but it is extremely rare during pregnancy with an estimated incidence of one per 10,000 to 15,000 deliveries [1]. Few cases are described in the literature, especially on its correlation with subsequent pregnancy.

Women with prolapse may have a variety of pelvic floor symptoms. Symptoms include pelvic heaviness, a dragging sensation in the vagina, protrusion coming down from the vagina and backache, but only some of these symptoms are directly related to the prolapse.

## Case presentation

A 36-year-old Caucasian woman, gravida 3, para 2, presented to our antenatal outpatient clinic in the 10^th ^week of gestation complaining of uterine prolapse and amenorrhea. Five years earlier, at the age of 31 years, she had her first spontaneous vaginal delivery, after 39 weeks of clinically unremarkable gestation and after a seven-hour labor. A living male baby weighing 2950 g, with Apgar scores of 10/10, was delivered. After that, a total uterine prolapse (POP-Q IV) was observed and, therefore, a pelvic reconstruction operation was scheduled. However, she missed the appointment and she was lost to follow-up.

Four years later, at the age of 35 years, the patient had her first pregnancy in a prolapsed uterus and the delivery was performed by an elective caesarean section after 38 weeks of gestation. During this second pregnancy follow-up she experienced symptoms of heaviness, but no pelvic pain or urinary incontinence. Pelvic examination showed that the uterus persisted in the pelvis because of increased volume. The cervical os was closed, while the entire cervix was lying outside the vulva during the first three months and after week 18 it appeared completely inside. When the cervix was outside the vulva, it appeared enlarged and edematous with marked ectropion but it was not ulcerated. A live male baby weighing 3150 g, with Apgar scores of 10/10, was delivered with elective caesarian section. After that, a total uterine prolapse persisted but she refused any procedure for pelvic reconstruction; neither was a vaginal pessary used.

**Figure 1:**
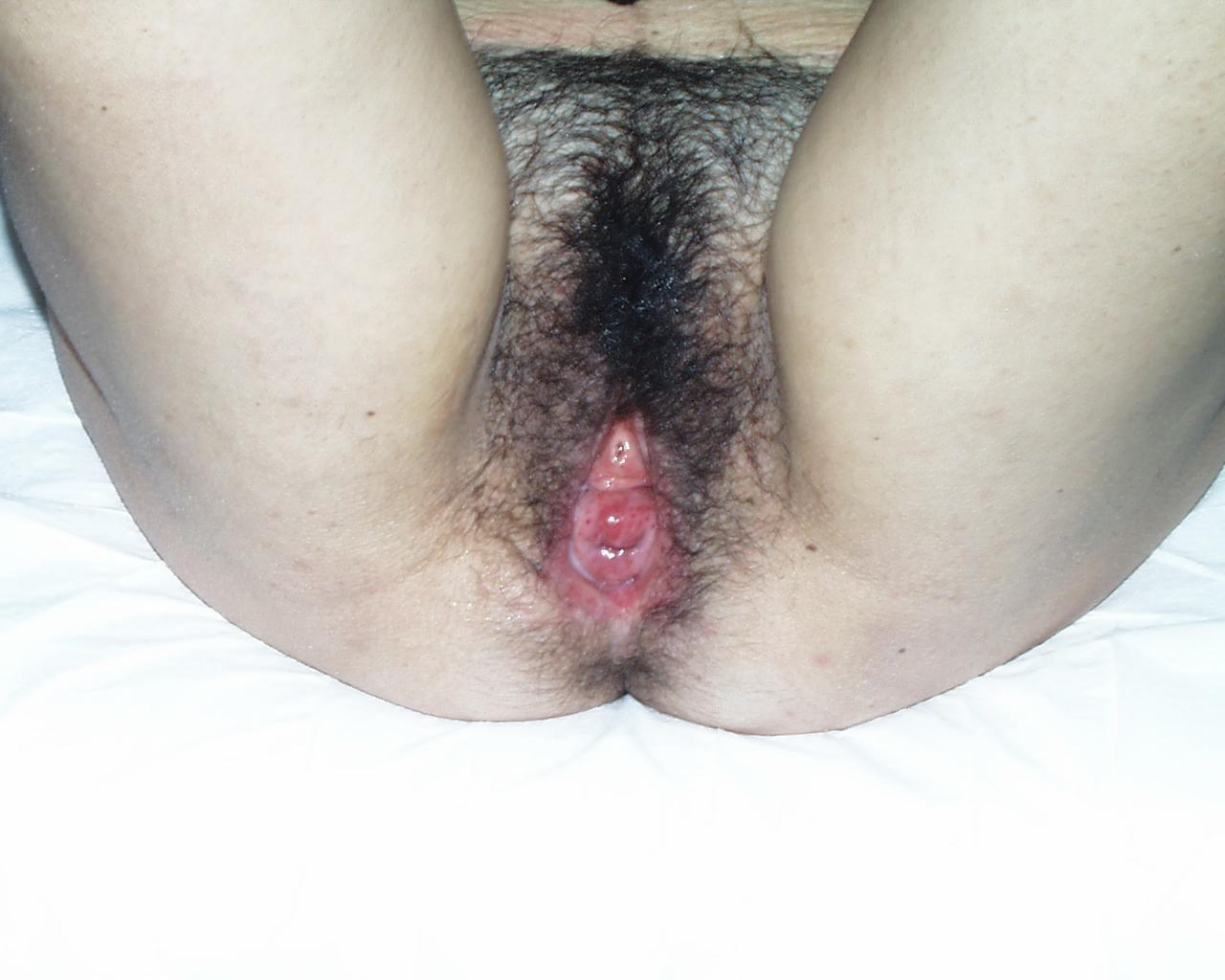
**Resolution of the prolapse during the final period of gestation because of the increased uterus volume**.

One year later, at the age of 36 years, she presented again in our clinic with a 10-week pregnancy in a prolapsed uterus. A vaginal pessary was applied to keep the uterus inside the pelvis after manual reposition. The pessary was removed at the 24^th ^week. The gravid uterus persisted in the abdominal cavity because it was increased in volume (Figure 1). She did not show any symptoms of heaviness or urinary incontinence. The cervix was lying at the os of the vulva (POP-Q II) without signs of dessication or ulceration. It was enlarged and edematous but showed no evidence of cervical incompetence.

Serial transabdominal ultrasonograpic examinations showed a normally developing fetus in longitudinal position in the uterine cavity. Elective caesarean section was performed at the 38^th ^week. A living, healthy female baby weighing 3030 g, with Apgar scores of 10/10, was delivered.

**Figure 2:**
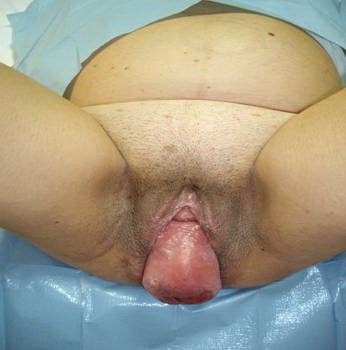
**The patient after elective Caesarean section with total uterine prolapse**.

The postnatal period was uneventful and she was discharged home four days later in good health. Normal postpartum uterine involution was observed. After that, a total uterine prolapse (POP-Q IV) was still observed (Figure 2).

She is scheduled for follow-up examination and pelvic reconstruction surgery.

## Conclusion

Uterine prolapse is a common gynecologic condition but is extremely rare during pregnancy as shown by the few similar reports in the literature. Certainly the literature before 1970, while it does not always specify the exact degree of prolapse, suggests a much higher incidence in more disadvantaged areas and where grand multiparity was more common. We found two reports of natural term pregnancy with an initially procidencia uteri [2,3] and one case of in vitro fertilization and embryo transfer pregnancy with an initially complete uterine prolapse [4].

In the classification of uterine prolapse using the POP-Q evaluation, total uterine prolapse extending outside the introitus with eversion of the entire vagina without standing or traction is called third-fourth degree prolapse [5].

Multiple factors are usually involved in the genesis of uterine prolapse but the most prominent cause is pregnancy, associated with prolonged labor, or difficult delivery. However, it may also occur spontaneously, although very rarely, even in nulliparous women.

In our case, the patient had sexual intercourse without any vaginal pessary.

Conservative management with close follow-up and bed rest can alleviate clinical symptoms and reduce potential complications correlated with this condition [2,4]. We recommend a vaginal pessary application during the first six months, until the volume of the uterus volume is increased

Complications such as patient discomfort, cervical dessication and ulceration, urinary tract infection, acute urinary retention, abortion, pre-term labor and even maternal death have been previously described [3,6]. We did not observe any of these complications except patient discomfort with light symptoms of heaviness without pelvic pain.

Although in a very recent report Eddib *et al*. [3] managed a similar case with a vaginal delivery, we believe that elective Caesarean section near term could be the safest delivery modality in order to avoid a progression of the prolapse and uterine rupture or damage [1,6]. This procedure can be also effective in preventing organ prolapse.

In conclusion, our case illustrates that natural pregnancy during uterine prolapse is possible and the management of uterine prolapse during labor should be individualized, depending on the severity of the prolapse, gestational age, parity, and the patient's preference.

A vaginal delivery can be expected, but, according to our experience, an elective caesarean section near term could be a valid and safe delivery option.

## Consent

Written informed consent was obtained from the patient for publication of this case report and accompanying images. A copy of the written consent is available for review by the Editor-in-Chief of this journal.

## Competing interests

The authors declare that they have no competing interests.

## Authors' contributions

DD analyzed and interpreted the patient data, performed clinical examinations and revised the manuscript. SG designed the case and was a major contributor in writing the manuscript. Both authors read and approved the final manuscript.
